# The Microscope in Medicine

**Published:** 1891-05-02

**Authors:** Frank J. Wethered


### THE MICROSCOPE IN MEDICINE.

By Frank J. Wethered, M.D.

### XIII.—EXAMINATION OF THE BLOOD.

The examination of the blood for clinical purposes does not yield such valuable information as does that of the sputum and urine. This is partly due to the great changes which occur in it in health, but, however, especial investigation in certain diseases may afford important corroboration of an otherwise merely presumptive diagnosis. The mode of obtaining a sample of blood for examination is very simple. The patient's finger is compressed towards the tip for a few moments and then pricked with a sharp surgical needle. A drop ought then to exude without any pressure. It is important to bear this in mind as, if the blood has to be forced out, the proportion between the corpuscular elements and the serum is materially altered.

One of the most common operations in connection with the examination of the blood is the estimation of its corpuscular value. This is usually accomplished by the "hæmacytometer." There are several forms of this apparatus, but the one generally used in this country is that devised by Dr. Gowers, and made by Hawksley. It consists of (1) a pipette which, when filled to the mark on its stem, holds exactly 995 cubic millimetres, and is provided with an india-rubber tube and mouthpiece; (2) a capillary tube graduated to five cubic millimetres; (3) a small glass jar in which the blood is diluted; (4) a glass stirrer; (5) a glass slide on which is arranged a cell one-fifth of a millimetre deep, the bottom being ruled in one-tenth millimetre squares. This slide is attached to a metal plate by means of springs which also serve to keep the coverglass in position. The estimation of the corpuscles is made as follows: A "diluting solution" is first prepared, having the following composition: Sulphate of soda, 104 grains; acetic acid, 1 drachm; distilled water, 4 ounces.

Nine hundred and ninety-five cubic millimetres of this solution are measured off by the pipette and placed in a mixing jar. A drop of blood is then obtained from the patient's finger by the method described above, and five cubic millimetres of it drawn up into the capillary tube, and then blown into the solution. The mixture is then thoroughly stirred with the glass rod and a small drop placed in the centre of the cell. The coverglass is gently put into position and secured by the two springs. The plate is then arranged under the microscope and the squares focussed with a ¼ inch lens. In a few minutes the corpuscles will have sunk to the bottom of the cell, and a variable number will be seen lying in each square. The number in ten squares is then counted and the sum multiplied by ten thousand will give the number of corpuscles in a cubic millimetre of blood.

The average number in normal blood is 5,000,000, this corresponding to about 50 in a square. Therefore, the number in two squares of the instrument will always represent the proportion of the corpuscular richness to normal blood.

It is often required to estimate the colourless corpuscles only. They are difficult to see, and generally bear a very small proportion to the red. One method of counting them is to ascertain the number of red corpuscles per square and then to count the number of squares in the field. If then the focus is raised so that the corpuscles are becoming indistinct, the white ones, from their higher refracting power, will appear like bright points, and the number in a series of fields can easily be counted. For example, the number of red corpuscles per square has been found to be 40, and the field contains 15 squares, that is 600 corpuscles per field. Ten fields contain 15 white corpuscles; the proportion of white to red will therefore be 1 to $\frac{600 \times 10}{15}$ = 1 to 400.

A rather more simple method is that introduced by Thoma. The blood is mixed with ten times its volume of 5 per cent. solution of acetic acid; this destroys all the red corpuscles, and consequently only the white remain in view.

The normal proportion of white to red cells is 1 to 300, but varies greatly even in health. Thus the white cells are greatly increased in number after a meal.

In conjunction with counting the corpuscles the richness of the blood in hæmoglobin is usually estimated. This is accomplished by another instrument introduced by Dr. Gowers, known as the hæmoglobinometer. It consists of two slender glass tubes, one of which contains a preparation of carmine and glycerine jelly coloured to represent a dilution of one part of healthy blood and 100 of water; the other tube being graduated into 100 divisions, each being equal to the volume of blood taken (20 cubic millimetres), so that 100 divisions equal 100 times the volume of blood. With these tubes a pipette is supplied graduated to hold 20 cubic millimetres. This quantity of blood is drawn up into the pipette and then placed in the graduated tube. Distilled water is then added drop by drop until the tint reaches that of the standard. The height of the diluted fluid is then read off, the number representing the amount of hæmoglobin; for example, a specimen of blood requires to be diluted up to the 50 mark on the tube before its tint corresponds to that of the standard. The blood examined therefore contains 50 per cent. of the normal quantity of hæmoglobin.

The conclusions to be drawn from the results of the two operations above described are important only in connection with certain conditions. The first of these is known as *Oligocythæmia*. By this is understood a diminition in the number of the cellular elements of the blood. As already stated the normal number of red blood corpuscles in a cubic millimetre of blood is 5,000,000. In disease this number may diminish temporarily or permanently to 2,000,000 or even sink as low as 360,000 per cubic millimetre. (Jaksch.)

Such a condition may occur as a consequence of hæmorrhage, either due to injury or disease. As a permanent state it may accompany any disease which is attended with deficient regeneration of blood. The reverse of the above condition, *i.e.*, an increase in the number of the cellular constituents has not been proved to occur, but the proportion between the red and the white may be considerably altered.

The normal proportion of red to white cells is one to three hundred, but this may be so altered that the white equal, or even exceed, the red in number.

The term *Leucocytosis* is applied to that condition in which the number of white corpuscles is temporarily much increased. This occurs physiologically after a meal, but much greater degrees are found in disease. Virchow states that whenever the lymphatic glands are affected this condition is produced.

When the white corpuscles are permanently increased the condition known as leukæmia is brought about. In exaggerated cases the blood has a greyish-pink, pus-like appearance, an acid reaction, and a peculiar mawkish odour. It is usual to classify *leucœmia* as splenic, lymphatic, and myelogenic according as the disease is seated in the spleen, lymphatic glands, or marrow of the bones. Ehrlich has lately made some remarkable experiments as regards the reaction of the white corpuscles to stains in this condition. There is, however, not space to describe here his investigations. Those interested in the matter may refer to the *Zeitschrift für Klinischer Medicin I.*, 553, 1880. In the condition now under consideration the red blood corpuscles often undergo changes of form. To this condition Quincke gave the name of *Poikilocytosis.*

Some important parasites are met with in the blood. The chief of these being the Filaria sanguinis hominis. In Europeans these seldom occur, but in the inhabitants of tropical countries, some of whom appear to be in good health, whilst others suffer from chyluria or elephantiasis, the embryos are often seen. They occur as small worm-like bodies, each being about $\frac{1}{90}$ of an inch in length. They are easily obtained by pricking the finger and placing the drop directly under a coverglass, but can only be found whilst the patient is asleep. The parent worm occupies the lymphatics, and consequently can only be discovered after death. It resembles, according to Manson, "a delicate thread of catgut, animated and wriggling."

Only a few of the vegetable parasites have been found in the blood during life, of these the most important is the tubercle bacillis, and the bacillis anthracis. The former has been occasionally met with in cases of miliary tuberculosis. Coverglass preparations of the blood are stained by Neelsen's method. (see "Sputum.") The anthrax bacilli are stained by Gram's method.

Though no cases of relapsing fever have been met with in England for many years, the spirillum which is constantly associated with the disease, has been demonstrated in past epidemics, and can be recognised without the aid of reagents. It is known as the "Spirillum Obermeieri," and takes the form of a delicate, homogeneous, spirally-twisted filament, and is often seen in active movement.

